# Editorial: Environmentally-responsive biomaterials for major diseases treatment

**DOI:** 10.3389/fbioe.2025.1604616

**Published:** 2025-04-25

**Authors:** Yan Zhao, Donglin Xia, Yao Luo

**Affiliations:** ^1^ Institute of Biomedical Health Technology and Engineering, Shenzhen Bay Laboratory, Shenzhen, China; ^2^ School of Public Health of Nantong University, Nantong, China; ^3^ Department of Laboratory Medicine, Sichuan Medical Laboratory Clinical Medicine Research Center, West China Hospital, Sichuan University, Chengdu, China

**Keywords:** biomedical materials, microenvironmental stimuli, disease treatment, injury repair, nanomedicine, targeted release, release mechanism

## 1 Introduction

Environmentally-responsive biomaterials are a novel class of materials that undergo structural or functional changes by altering their internal atom arrangement in response to environmental stimuli. These stimuli include endogenous disease microenvironments, such as the acidic pH of tumor microenvironments or elevated enzymatic activity in inflamed tissues, external physical triggers, such as temperature, light, ultrasound, or magnetic fields, and the combinations of these stimuli (Mura et al., 2013; Chen et al., 2022; Zhu et al., 2023). A variety of environmentally-responsive biomaterials have been developed to date, including DNA nanostructures (Zhao et al., 2023; Li et al., 2024; Zhao et al., 2024c; Zhao et al., 2021; Liu et al., 2023; Ji et al., 2022), hydrogels (Ma et al., 2023; Zhao et al., 2024b; Guo et al., 2023), nanomicelles (Wang et al., 2024; Uthaman et al., 2024; Wang et al., 2023) and biomembranes-based materials (Su et al., 2022). Those environmentally-responsive biomaterials achieve the delivery and release of therapeutics with precise spatial and temporal control, minimizing off-target effects and maximizing treatment efficacy *in vitro* and *in vivo* (Xue et al., 2023; Cui et al., 2024; Wei et al., 2011; Xia et al., 2019).

For the treatment of clinical diseases, one of the primary advantages of environmentally-responsive biomaterials is their ability to provide targeted and on-demand therapeutic solutions. By leveraging the unique pathological microenvironments of diseases or physical stimuli, these materials enhance the selectivity and efficiency of treatments. Additionally, their adaptability reduces the need for external intervention, improving patient compliance and reducing systemic toxicity. Applications of environmentally-responsive biomaterials span across major disease treatments (Zhao L. et al., 2024; Guo et al., 2016; Duan et al., 2022; Xia et al., 2020), including cancer, diabetes, cardiovascular disorders. For example, pH-sensitive hydrogels are used for localized drug delivery in cancer therapy (Lin et al., 2025), while thrombin-responsive DNA nanomachines aid in precise delivery and accurate dosing of tissue plasminogen activator release for thrombolytic therapy (Yin et al., 2024). Furthermore, advancements in this field are paving the way for tissue engineering (Zhang et al., 2018), regenerative medicine, and autoimmune diseases (Shodeinde et al., 2020), driving a paradigm shift in modern healthcare (Zhang et al., 2015). In summary, environmentally responsive biomaterials embody the intersection of material science and medicine, offering innovative and sustainable solutions to some of the most pressing challenges in disease treatment.

This Research Topic collected excellent works on the “Environmentally-responsive biomaterials for major diseases treatment,” and a total of 9 articles from 38 authors were accepted. The contributions have deepened the understanding of this Research Topic from perspectives such as the development of environmentally-responsive biomaterials and the strategies for utilizing environmental signals in clinical treatment. This Research Topic can be broadly divided into the following three subfields.

## 2 External physical triggers

External stimuli, such as temperature, light, ultrasound, and magnetic fields, enable precise, controllable activation of biomaterials, allowing for non-invasive and on-demand therapeutic interventions. In our Research Topic, Zhang et al. developed an optogenetic-based mitochondrial aggregation system (Opto-MitoA) based on a CRY2clust/CIBN light-sensitive module. Through rapidly controlling mitochondrial aggregation in cells upon blue light illumination, this system could increase the energy-generating function of mitochondria and alleviate niclosamide-caused cell dysfunction. Chen et al. synthesized albumin-loaded Tanshinone IIA and near-infrared photothermal agent IR780 nanoparticles for managing chronic and infected wounds. The release of Tanshinone IIA was improved under laser irradiation, thus realizing enhanced wound healing. He et al. reviewed polydopamine-coated metal-organic frameworks (MOFs@PDA) multifunctional nanomaterial, highlighting their potential in cancer therapy. By leveraging strong photothermal responsiveness of polydopamine, MOFs@PDA enable controlled drug release triggered by near-infrared light, enhancing therapeutic precision and minimizing side effects in cancer treatment.

## 3 Endogenous disease microenvironments

Endogenous disease microenvironments, including acidic pH, hypoxia, and enzymatic activity, provide intrinsic biological triggers that enable biomaterials to achieve site-specific responses and targeted drug release. Bin et al. designed GSH-responsive nanomicelles that release glucose transporter 1 (GLUT1) inhibitor to block mononuclear phagocyte system (MPS) uptake, significantly improving tumor treatment. Chu et al. emphasized the critical role of rebuilding the myocardial microenvironment in mesenchymal stem cells (MSCs)-based myocardial regeneration. This review highlights the strategies for promoting angiogenesis to improve MSCs survival and function in the treatment of ischemic heart disease. Wu et al. developed a multiplexed microfluidic immunoassay chip based on nanozyme technology for detecting eight respiratory viruses, demonstrating its application in sensing endogenous microenvironments through virus-specific antigen detection. Zhang et al. reviewed reactive oxygen species (ROS)-responsive biomaterials for Myocardial ischemia-reperfusion injury (MIRI) treatment as the ROS microenvironment. They systematically summarized the fabrication strategies and therapeutic platforms of ROS-responsive biomaterials, paving the way for their clinical translation. Han et al. utilized nanodiamonds as carriers to deliver MicroRNA-7 into dopaminergic neurons for the treatment of Parkinson’s disease. They used the nanodiamonds & MicroRNA-7 complex (N-7) to inhibit the expression of αsynuclein, reduce oxidative stress and restore dopamine levels effectively.

## 4 Multiple stimuli combinations

The combination of multiple stimuli integrates the advantages of both external and endogenous triggers, enhancing the specificity, efficiency, and adaptability of biomaterials in complex disease treatments. Xie and Xie reviewed the controlled drug release enabled by physical-, chemical-, biological- and multiple-stimuli-responsive hydrogels and their applications in treating brain disease. They propounded that a multidisciplinary approach that combines expertise from various fields is critical, will greatly enhance scientific research, and will ultimately lead to new treatment options for patients.
